# Quantitative Measurement of Elasticity of the Appendix Using Shear Wave Elastography in Patients with Suspected Acute Appendicitis

**DOI:** 10.1371/journal.pone.0101292

**Published:** 2014-07-22

**Authors:** Seung-Whan Cha, Ik Yong Kim, Young Wan Kim

**Affiliations:** 1 Department of Radiology, Wonju College of Medicine, Yonsei University, Wonju, Korea; 2 Department of Surgery, Wonju College of Medicine, Yonsei University, Wonju, Korea; The University of Queensland, Australia

## Abstract

**Introduction:**

Shear wave elastography (SWE) has not been studied for diagnosing appendicitis. We postulated that an inflamed appendix would become stiffer than a normal appendix. We evaluated the elastic modulus values (EMV) by SWE in healthy volunteers, patients without appendicitis, and patients with appendicitis. We also evaluated diagnostic ability of SWE for differentiating an inflamed from a normal appendix in patients with suspected appendicitis.

**Materials and Methods:**

Forty-one patients with clinically suspected acute appendicitis and 11 healthy volunteers were prospectively enrolled. Gray-scale ultrasonography (US), SWE and multi-slice computed tomography (CT) were performed. The EMV was measured in the anterior, medial, and posterior appendiceal wall using SWE, and the highest value (kPa) was recorded.

**Results:**

Patients were classified into appendicitis (n = 30) and no appendicitis groups (n = 11). One case of a negative appendectomy was detected. The median EMV was significantly higher in the appendicitis group (25.0 kPa) compared to that in the no appendicitis group (10.4 kPa) or in the healthy controls (8.3 kPa) (p<0.001). Among SWE and other US and CT features, CT was superior to any conventional gray-scale US feature or SWE. Either the CT diameter criterion or combined three CT features predicted true positive in 30 and true negative in 11 cases and yielded 100% sensitivity and 100% specificity. An EMV of 12.5 kPa for the stiffest region of the appendix predicted true positive in 28, true negative in 11, and false negative in two cases. The EMV (≥12.5 kPa) yielded 93% sensitivity and 100% specificity.

**Conclusion:**

Our results suggest that EMV by SWE helps distinguish an inflamed from a normal appendix. Given that SWE has high specificity, quantitative measurement of the elasticity of the appendix may provide complementary information, in addition to morphologic features on gray-scale US, in the diagnosis of appendicitis.

## Introduction

Acute appendicitis is a common abdominal emergency requiring surgery. Diagnostic imaging modalities such as abdominal ultrasonography (US) and computed tomography (CT) have led to a decrease in the perforation rate [1], [2]. US is safe and widely available; however, diagnostic accuracy is sonographer-dependent. An effective US examination is difficult in obese patients, as fat absorbs ultrasound. CT is less operator-dependent and is useful for distinguishing other causes of abdominal pain. The disadvantages of CT are radiation exposure and contrast-induced hypersensitivity reactions. The choice of these studies depends upon patient factors such as age, obesity or pregnancy, and institutional factors such as the availability of US and CT examinations [3], [4]. In a recent meta-analysis, sensitivity and specificity of US were 78% and 83%, and sensitivity and specificity of CT were 91% and 90%, respectively [5]. CT shows higher sensitivity and specificity but the diagnostic accuracies of both studies are acceptable for diagnosing appendicitis. Magnetic resonance imaging (MRI) is emerging as a promising modality, as MRI is operator-independent and avoids radiation exposure and the use of contrast agent. Orth et al. [6] showed that the diagnostic performance of nonenhanced MRI was comparable to that of US in pediatric patients. Aspelund et al. [7] demonstrated that a radiation-free imaging pathway such as selective MRI after US was comparable to a primary CT strategy in terms of time to appendectomy, negative appendectomy rate, and perforation rates in pediatric patients. Leeuwenburgh et al. [8] found that the diagnostic accuracies of conditional MRI following US or immediate MRI were comparable to those of conditional CT following US in adult patients with suspected appendicitis.

Ultrasound elastography noninvasively assesses the elastic properties of tissue. The technical concept is that tissues are stressed by mechanical forces and the amount of tissue displacement in response to these forces is measured with ultrasound [9]. Ultrasound elastography has gained popularity for distinguishing malignant nodules from benign nodules in patients with breast and thyroid diseases [10], [11]. Malignant nodules are stiffer than the normal parenchyma [12]. Several methodologies have been introduced to measure tissue hardness. First, strain elastography measures the degree of tissue displacement induced by the operator's external compression. Elasticity is expressed as a strain ratio or determined by semiquantitative scoring systems [10]. Second, acoustic radiation force impulse elastography measures tissue displacement signals generated by short-duration acoustic impulses. This method provides quantitative information using shear wave velocity. Tissue stiffness is expressed on contrasted images without a color-coded map [13]. Third, shear wave elastography (SWE) has been recently introduced. One major advantage over strain elastography is that this method does not require manual compression. Pulses from the ultrasound probe stimulate the target tissue and an ultrafast ultrasound scanning system captures shear wave propagation with plane waves at acquisition speeds of up to 20,000 hertz (Hz). Tissue elasticity is determined by shear wave velocity or kilopascals, which is the International System of Units derived unit of pressure. The elastic property is expressed by a blue color in softer tissue and red in harder tissue [14].

SWE is radiation-free and easy to perform in addition to conventional gray-scale US [9]–[14]. To date, SWE has not been studied for diagnosing appendicitis. We hypothesized that an inflamed appendix would be stiffer than that of a normal appendix. Thus, we aimed to evaluate the difference in elastic modulus values of the appendix among healthy volunteers, patients with no appendicitis, and patients with appendicitis. We also evaluated the diagnostic ability of SWE for distinguishing an inflamed from a normal appendix in patients with suspected appendicitis.

## Materials and Methods

### Ethics statement

All clinical investigations were conducted according to the principles expressed in the Declaration of Helsinki. This study was registered with the Clinical Research Information Service, Korea Centers for Disease Control and Prevention (http://cris.cdc.go.kr) (KCT0000764). This study was approved by the Institutional Review Board (IRB) at Yonsei University Wonju Severance Hospital (CR213002–003).

### Patients and healthy controls

**Figure 1:**
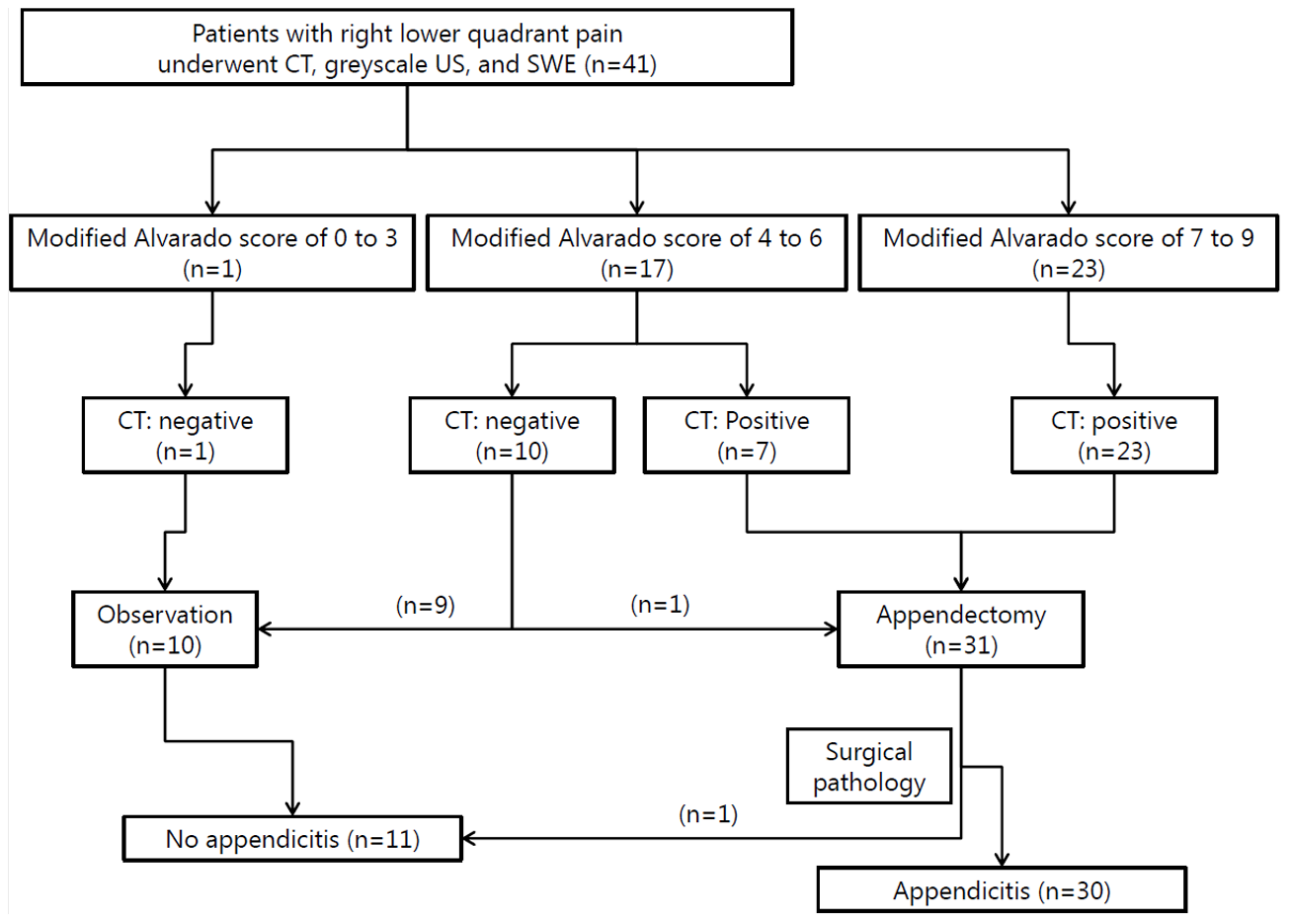
Patient enrollment (n = 41). Only the modified Alvarado scores and the CT results were considered during decision making for appendectomy. Positive CT results were defined when three radiologic features (≥6 mm in diameter, enhancement of periappendiceal fat, and wall thickening) were seen at the same time. CT, computed tomography; US, ultrasonography; SWE, shear wave elastography.

Forty-seven patients (age, ≥20 years) with clinically suspected acute appendicitis were prospectively enrolled in this study between June 2013 and March 2014. After enrollment, four patients with a periappendiceal abscess and two patients who withdrew consent were excluded. Patient recruitment is illustrated in Figure 1. Written informed consent was obtained from all participants, and consenting patients underwent both SWE and CT. Two colorectal surgeons (IYK and YWK with >10 years experience) made all decisions to perform an appendectomy based on the modified Alvarado scoring system and CT findings [15]. According to the IRB's recommendation, gray-scale US and SWE findings were not considered during the treatment decision making process because CT is a routine imaging study in patients with abdominal pain at our institution. The modified Alvarado score (a total of 9 points) was calculated as follows; migratory right lower quadrant pain (1 point), anorexia (1 point), nausea/vomiting (1 point), tenderness in the right lower quadrant area (2 points), rebound tenderness in the right lower quadrant area (1 point), fever >37.5°C (1 point), and leukocytosis (2 points). Positive CT imaging was defined when patients had all three CT features such as enlarged appendiceal diameter >6 mm, appendiceal wall thickening, and appendiceal wall enhancement. We excluded pregnant women, patients with a CT contrast-dye allergy, patients with known inflammatory bowel disease, patients with severe comorbid conditions or those with a suspicious periappendiceal abscess.

Eleven healthy volunteers (age, ≥20 years) without abdominal pain were included prospectively. Written informed consent was obtained from all participants, and consenting individuals underwent SWE.

### Study design

The hypothesis was that an inflamed appendix would lose elasticity due to acute inflammation. The primary endpoint of this study was to evaluate the difference in elastic modulus values of the appendix among healthy volunteers, patients with no appendicitis, and patients with appendicitis. The secondary endpoint was to evaluate the diagnostic ability of SWE for distinguishing an inflamed from a normal appendix in patients with suspected appendicitis.

### CT examination

The CT examination was performed using a multi-slice CT scanner (Brilliance CT 64-channel scanner, Philips, Cleveland, OH, USA). Intravenous contrast (120 cc) was administered 70 seconds prior to the scan. Serial 3-mm axial images were obtained from the diaphragm through the perineum. The CT report included >6 mm diameter, enhancement of periappendiceal fat, inflammatory thickening of the appendiceal wall, or presence of appendicolith. Two radiologists (SYC and KSK) interpreted the CT examinations.

### Gray-scale US and SWE examination

After the CT examination, one radiologist (SWC, >15 years experience with gastrointestinal radiology) who was unaware of the CT findings performed gray-scale US and SWE. The Aixplorer US system (SuperSonic Imagine S.A., Aix-en-Provence, France) was used with a convex broadband probe.

First, gray-scale US was performed with the patient in the supine position. The graded compression technique was used to obtain conventional longitudinal and transverse scan images. After identifying the vermiform appendix, the radiologist recorded gray-scale US features such as >6 mm diameter, echogenicity of periappendiceal fat, inflammatory thickening of the appendiceal wall, or presence of appendicolith.

Then, SWE was performed using the default device settings with respect to acoustic impulse intensity, smoothing factor, persistence, frame rate (7 frames/second), and display range of elastic modulus values (0–180 kPa). The patient held their breath for 5 seconds, and cine loops were captured. The cine loops were replayed until the color-coded elasticity reached a steady state. Blue and red on the SWE color map indicated low (soft) and high kPa (stiff), respectively. The radiologist selected a single SWE frame by visual inspection. A round region of interest (ROI) 1 or 2 mm in diameter was placed in the anterior, medial, and posterior appendiceal walls. The SWE software automatically quantified elastic modulus values (Q-Box), such as the maximum value (max Q-Box), minimum value (min Q-Box), mean value (mean Q-Box), and standard deviation. The radiologist repeated procedures such as capturing the cineloop, selecting an appropriate static image, and measuring the elastic modulus scale using ROI placement until he judged that elastic modulus values in the appendiceal walls were valid and reliable.

**Figure 2:**
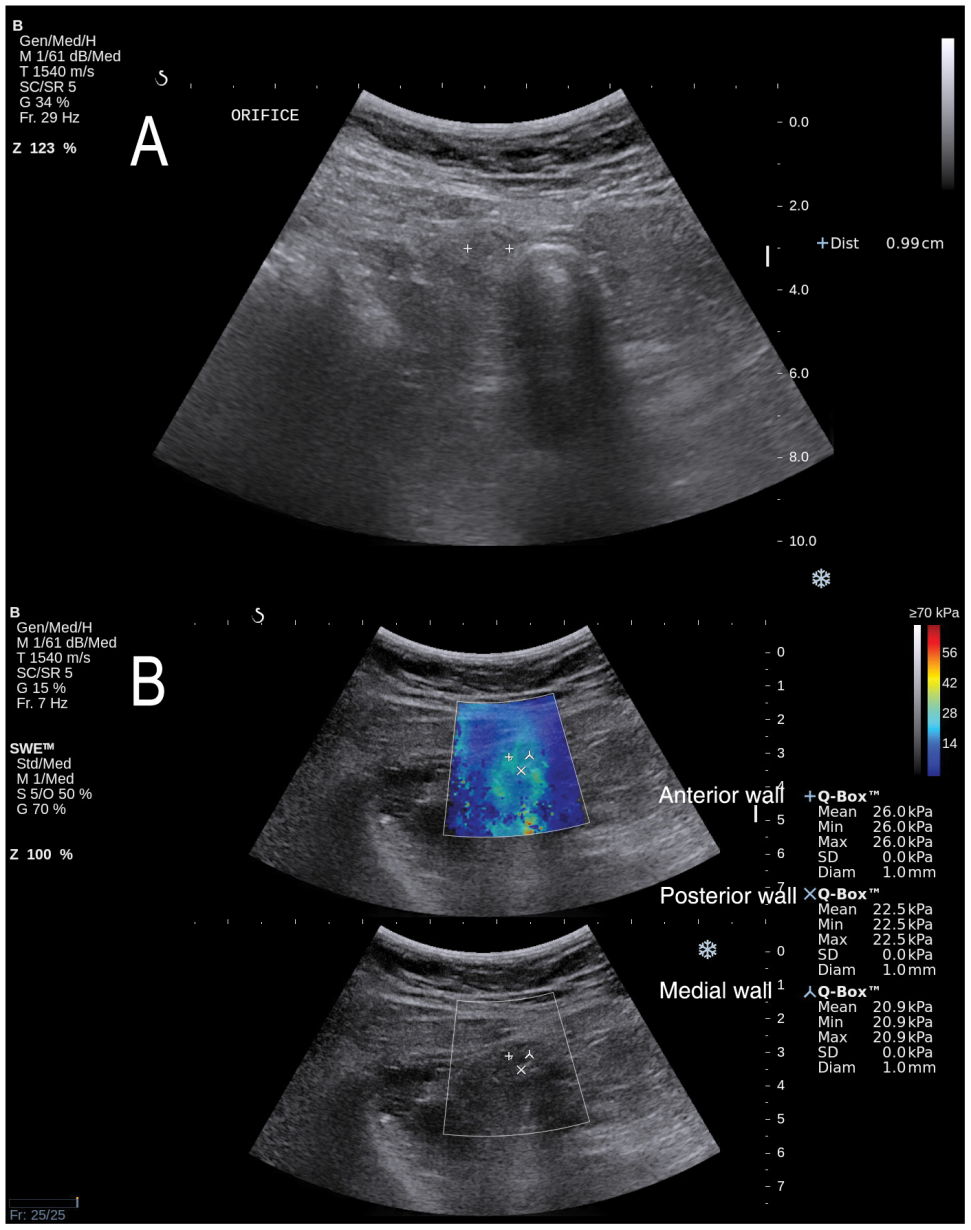
Gray-scale ultrasonography and shear wave elastography in a 47-year old female patient with appendicitis. A. Gray-scale ultrasonography was 9.9 mm in diameter. The echogenicity of periappendiceal fat and the appendiceal wall thickening were also noted (not shown). B. Elastic modulus scales (mean Q-Box) by shear wave elastography were 26.0, 20.9, and 22.5 kilopascal (kPa) in the anterior, medial, and posterior wall of the appendix, respectively. The highest elastic modulus scale (26.0 kPa) was selected for analysis. The modified Alavarado score of this patient was 7, and computed tomography showed a larger diameter (≥6 mm), enhancement of periappendiceal fat and wall thickening. The histopathology result showed appendicitis.

**Figure 3:**
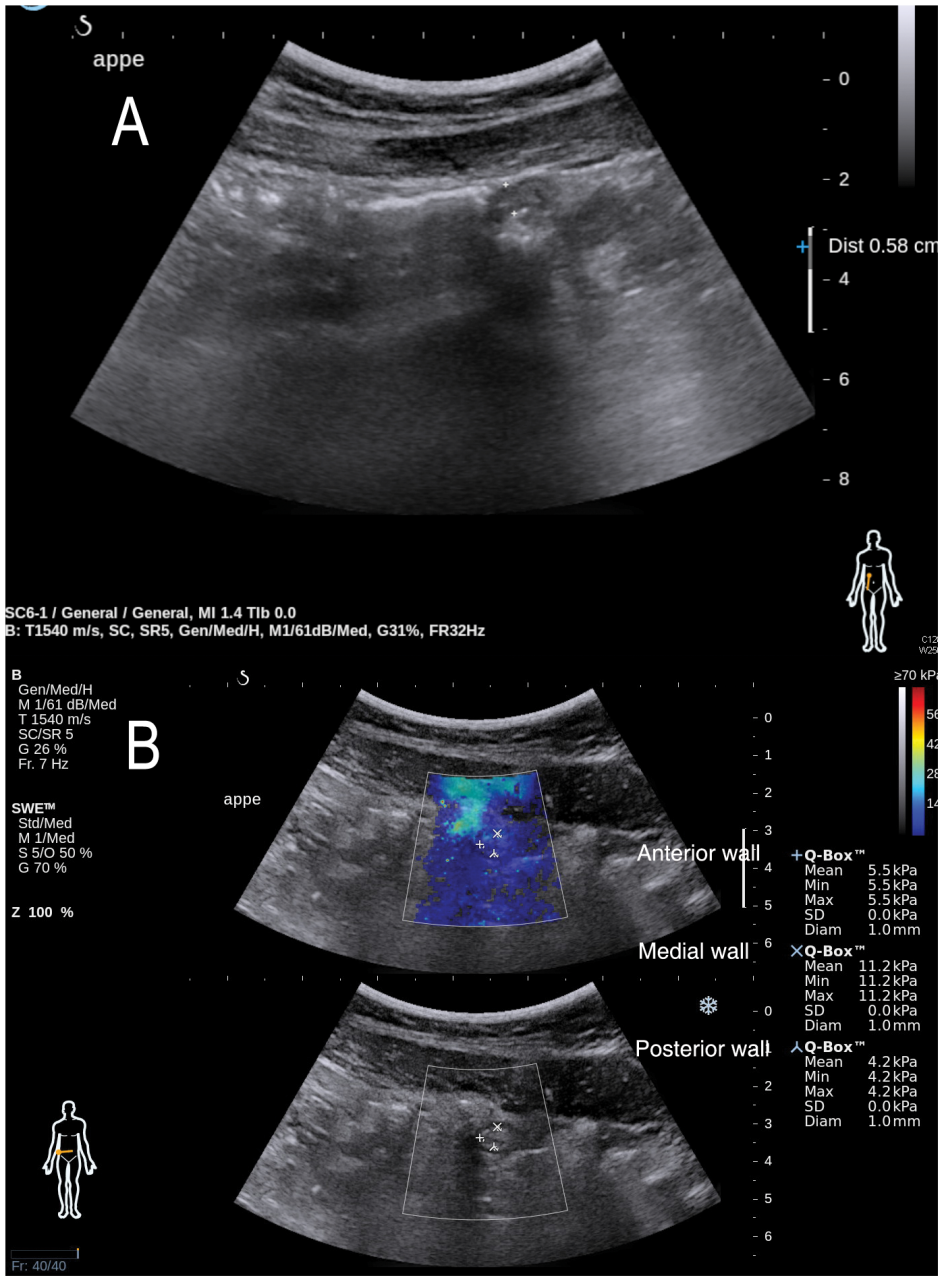
Gray-scale ultrasonography and shear wave elastography in a 24-year-old male patient with no appendicitis. A. Gray-scale ultrasonography was 5.8 mm in diameter. No echogenicity of periappendiceal fat or appendiceal wall thickening were noted. B. Elastic modulus scales (mean Q-Box) by shear wave elastography were 5.5, 11.2, and 4.2 kilopascal (kPa) in the anterior, medial, and posterior wall of the appendix, respectively. The highest elastic modulus scale (11.2 kPa) was selected for analysis. The modified Alavarado score in this patient was 5, and computed tomography showed a smaller diameter (<6 mm), no enhancement of periappendiceal fat, and the absence of appendiceal wall thickening. This patient was diagnosed with terminal ileitis and recovered without surgical intervention.

Young's elastic modulus scale (kPa) expressed by SWE was used as the main outcome measure of tissue elasticity. As shown in Figures 2 and 3, mean Q-Box was selected as an elastic modulus value for single ROI placement. Then, elastic modulus values (mean Q-Box) of the anterior, medial, and posterior appendiceal walls were compared, and the highest value was selected as the representative elastic modulus value of the appendix and used for final analysis.

### Sample size calculation

The sample size calculation was performed using PASS 2008 ver. 8.0 (NCSS, LLC, Kaysville, UT, USA). No study has evaluated elastic modulus values in the inflamed and normal appendix. One study evaluating liver stiffness by SWE showed that the mean elastic modulus value was 5.4±1.2 kPa in a nondiseased liver group and 8.1±3.0 kPa in a noncirrhotic chronic liver disease group [16]. Thus, our sample size was calculated using these results. The null hypothesis of this study was that there is no difference in mean elastic modulus values between an inflamed appendix and normal appendix. Group sample sizes of 13 and 13 to achieve 81% power to detect a difference of 2.7 between the null hypothesis that both group means are 8.1 and the alternative hypothesis that the mean of group 2 is 5.4 with estimated group standard deviations of 3.0 and 1.2 and with a significance level of 0.05 using a two-sided two-sample *t*-test [17], [18]. The final sample size was 30 patients when we considered a 10% drop rate.

### Statistical analysis

All statistical analyses were performed using IBM SPSS Statistics for Windows, ver. 20.0 (IBM, Armonk, NY, USA). The Mann–Whitney *U*-test was used to compare continuous variables and the chi-square test (Fisher's exact test) was used to compare categorical variables. Sensitivity, specificity, positive predictive value (PPV), and negative predictive value (NPV) were calculated for SWE and CT in patients with suspected appendicitis. A p-value <0.05 was considered statistically significant.

## Results

### Patient characteristics

Forty-one patients with right lower quadrant pain were enrolled. Their median age was 39 years, and 18 were men. Based on the modified Alvarado score and CT findings, 31 cases of appendectomy were performed and one case of a negative appendectomy was detected. Final diagnoses were as follows: appendicitis (n = 30), terminal ileitis (n = 5), irritable bowel syndrome (n = 2), acute gastroenteritis (n = 3), and diverticulitis (n = 1). All enrolled patients were classified into the appendicitis (n = 30) or no appendicitis groups (n = 11). Eleven healthy volunteers were also analyzed. Their median age was 36 years, and 10 were men.

No differences in age, gender, body mass index (BMI), white blood cell count, or neutrophil count were observed when comparing patients with appendicitis and without appendicitis. The median elastic modulus value of the appendix was significantly higher in patients with appendicitis (25.0 kPa) than that in patients without appendicitis (10.4 kPa) (p<0.001). No differences in age, BMI, white blood cell count, or neutrophil count were observed between patients with appendicitis and healthy controls. Men were more common in healthy controls (p = 0.004). The median elastic modulus value of the appendix was significantly higher in patients with appendicitis (25.0 kPa) than that in healthy controls (8.3 kPa) (p<0.001). No difference in the median elastic modulus value of the appendix was observed between patients without appendicitis (10.4 kPa) and healthy controls (8.3 kPa) (p = 0.3). Detailed patient characteristics are presented in Table 1.

**Table 1:** Patient characteristics.

		RLQ pain			No RLQ pain	
		Appendicitis (n = 30)	No appendicitis (n = 11)		Healthy controls (n = 11)	
		Median (IQR)	Median (IQR)	P	Median (IQR)	P*
Age (years)		42(29–53)	38(24–46)	1.0	36(29–39)	0.4
Gender, n(%)	Male	12(40)	6(54)	0.4	10(91)	0.004
	Female	18(60)	5(46)		1(9)	
Body mass index (kg/m^2^)		23.5(20.2–26.4)	23.8(22.2–25.2)	1.0	23.3(22.6–25.2)	1.0
White blood cell count (10^9^/L)		11.9(9.1–15.9)	11.1(8.6–12.1)	0.5	NA	
Neutrophil count (10^9^/L)		10.3(6–11.7)	7.3(6.3–9.6)	0.4	NA	
Elastic modulus scale (kPa) on shear wave elastography		25(18.1–32.8)	10.4(5.1–11.9)	<0.001	8.3(4.7–9.3)	<0.001

RLQ, right lower quadrant; IQR, interquartile range; NA, not available; kPa, kilopascal.

*Compared with patients with appendicitis.

### Imaging results compared with final diagnosis

When the three gray-scale US features such as >6 mm diameter, echogenicity of periappendiceal fat, and wall thickening were combined, 30, nine, and two cases were true positive, true negative, and false positive, respectively. The elastic modulus value (kPa) is displayed as a continuous variable, and we chose 12.5 kPa as the cutoff, which yielded the highest diagnostic ability. Using a cutoff of ≥12.5 kPa, 28, 11, and two cases were true positive, true negative, and false negative, respectively. When all three CT features such as enlarged appendiceal diameter >6 mm, appendiceal wall thickening, and appendiceal wall enhancement were combined, 30 and 11 cases were true positive and true negative, respectively. Other detailed results are presented in Table 2.

**Table 2:** Imaging results compared with final diagnosis.

		Appendicitis (n = 30)	No appendicitis (n = 11)
**Gray-scale ultrasonography**			
Diameter (≥6 mm)	Present	30	2
	Absent	0	9
Echogenicity of periappendiceal fat	Present	30	2
	Absent	0	9
Thickening of appendiceal wall	Present	30	3
	Absent	0	8
Appendicolith	Present	10	0
	Absent	20	11
Diameter (≥6 mm), echogenicity of periappendiceal fat, thickening of appendiceal wall	Present (all)	30	2
	Absent (one or more)	0	9
**Shear wave elastography**			
Elastic modulus scale (kilopascal)	≥12.5 kPa	28	0
	<12.5 kPa	2	11
**Computed tomography**			
Diameter (≥6 mm)	Present	30	0
	Absent	0	11
Enhancement of periappendiceal fat	Present	30	3
	Absent	0	8
Thickening of appendiceal wall	Present	30	2
	Absent	0	9
Appendicolith	Present	10	0
	Absent	20	11
Diameter (≥6 mm), echogenicity of periappendiceal fat, thickening of appendiceal wall	Present (all)	30	0
	Absent (one or more)	0	11

### Diagnostic ability of gray-scale US, SWE and CT

The gray-scale US data showed that the sensitivity of diameter (≥6 mm), echogenicity of periappendiceal fat, and wall thickening was 100%, 100%, and 100%, respectively. Specificity of diameter (≥6 mm), echogenicity of periappendiceal fat, and wall thickening was 82%, 82%, and 82%, respectively. When these three US features were combined, sensitivity was 100% and specificity was 82%.

The SWE results using a cutoff of ≥12.5 kPa showed that sensitivity and specificity were 93% and 100%, respectively. To find optimal cutoff values of SWE for diagnosing acute appendicitis, receiver operating characteristic (ROC) curve analysis was performed. The area under an ROC curve (AUC) represents the accuracy of a diagnostic test. AUC values range from 0.5 (no diagnostic ability) to 1.0 (perfect diagnostic ability). Using different cutoff values, the AUC values of 12.5 kPa criterion was the highest (0.967). Thus, we chose 12.5 kPa as the cutoff in this study.

The CT data showed that the sensitivity of diameter (≥6 mm), enhancement of periappendiceal fat, and wall thickening on CT was 100%, 100%, and 100%, respectively. The specificity of diameter (≥6 mm), enhancement of periappendiceal fat, and wall thickening on CT was 100%, 73%, and 83%, respectively. When the three CT features were combined, sensitivity was 100% and specificity was 100%. The sensitivity, specificity, PPV, and NPV values are presented in Table 3.

**Table 3:** Diagnostic ability of gray-scale ultrasonography, shear wave elastography, and computed tomography in patients with suspected appendicitis.

	Sensitivity (%)	Specificity (%)	PPV (%)	NPV (%)
**Gray-scale ultrasonography**				
Diameter (≥6 mm)	100	82	94	100
Echogenicity of periappendiceal fat	100	82	94	100
Thickening of appendiceal wall	100	73	91	100
Appendicolith	33	100	100	35
Diameter (≥6 mm), Echogenicity of periappendiceal fat, thickening of appendiceal wall	100	82	94	100
**Shear wave elastography**				
Elastic modulus scale (≥12.5 kPa)	93	100	100	85
**Computed tomography**				
Diameter (≥6 mm)	100	100	100	100
Enhancement of periappendiceal fat	100	73	91	100
Thickening of appendiceal wall	100	82	94	100
Appendicolith	33	100	100	35
Diameter (≥6 mm), enhancement of periappendiceal fat, thickening of appendiceal wall	100	100	100	100

PPV, positive predictive value; NPV, negative predictive value; kPa, kilopascal.

## Discussion

Multidetector CT has become the preferred imaging modality to diagnose appendicitis due to high sensitivity and specificity. MRI has been increasingly studied in recent years, as it has clear benefits in terms of not requiring a contrast agent and no radiation risk. In addition, accuracy of MRI was comparable to CT in the diagnosis of appendicitis in pediatric and adult populations [6]–[8]. However, US remains an essential tool for children and patients of childbearing age. Thus, we explored whether SWE could be used to improve diagnostic performance of conventional gray-scale US. The advantage of SWE is that it enables quantification of tissue elasticity using the elastic modulus scale, which is objective information on a given target tissue. In addition, conventional gray-scale US images can be obtained using a SWE system [14], [19].

The primary endpoint was to evaluate the difference in elastic modulus values of the appendix among healthy volunteers, patients without appendicitis, and patients with appendicitis. It was possible to measure appendiceal elasticity using elastic modulus values in patients with suspected appendicitis as well as in healthy volunteers. SWE captures the speed of sheer wave propagation and the displacement response from stimulated tissue. Higher shear wave speeds and smaller displacement are seen in stiffer tissue. The major finding of this study was that the median elastic modulus value of an inflamed appendix was higher than that of a normal appendix, which was compatible with the hypothesis of this study. In addition, no difference in the median elastic modulus value of the appendix was observed between patients without appendicitis and healthy controls.

The secondary endpoint was to evaluate the diagnostic ability of the imaging modalities. We confirmed that CT was superior to any conventional gray-scale US feature. Using either the CT diameter criterion or the three combined CT features, 30 and 11 cases were true positive and true negative, respectively. The elastic modulus value (≥12.5 kPa) revealed that 28, 11, and two cases were true positive, true negative, and false negative, respectively. An elastic modulus value of 12.5 kPa for the stiffest region of the appendix exhibited 93% sensitivity, and 100% specificity. Specificity of SWE was higher than that of other gray-scale US features such as >6 mm diameter, echogenicity of periappendiceal fat, and wall thickening. Due to the high specificity, SWE may be suitable for distinguishing an inflamed from a normal appendix independent of gray-scale US features. Although this study yielded 100% specificity, caution should be used when interpreting the results due to the limited sample size.

Negative appendectomy rates are 2.7–15.5% in the literature [20], [21] and we found one case of a negative appendectomy (1/31, 3%) in this study. That patient had a low elastic modulus value (<12.5 kPa) on SWE and a smaller diameter (<6 mm) on CT scan. However, other US (≥6 mm in diameter, echogenicity of periappendiceal fat, wall thickening) and CT features (enhancement of periappendiceal fat and wall thickening) suggested appendicitis. However, the histopathological results after the appendectomy showed a normal appendix.

Several factors may complicate accurate measurement of appendix elasticity. In particular, an anteriorly located cecum in the retrocecal appendix may produce an unnecessarily displaced signal. Shear waves generated by supersonic source are attenuated during propagation. Although we used a convex probe (Supercurved, bandwith: 1–6 MHz), the effective depth of penetration seemed to be limited to 2–3 cm. This might be the reason for the two false-negative cases in this study. One study that evaluated solid breast masses showed that a deeply located lesion is a risk factor for false-positive or false-negative SWE results [22]. In addition, the inability to identify the appendix, which is a major limitation of US, may limit the use of SWE. However, when the appendix is found, SWE could improve the accuracy of gray-scale US.

The limitation of this study is the small sample size; however, this is the first prospective study to evaluate diagnostic ability of SWE in patients with suspected appendicitis. We excluded patients with a periappendiceal abscess, as this condition may accompany a gangrenous change in the appendix. Gangrenous appendiceal tissue may become softer. Future studies should be directed toward validating the correlation between SWE and surgical pathology. In addition, reliable and reproducible cutoff values need to be defined.

In summary, patients with an inflamed appendix (25.0 kPa) showed significantly higher median elastic modulus values than patients with a normal appendix (10.4 kPa) or healthy volunteers (8.3 kPa). These results suggest that SWE helps distinguish an inflamed from a normal appendix. Using a cutoff of 12.5 kPa, SWE yielded 93% sensitivity and 100% specificity in patients with suspected appendicitis. Given that SWE has high specificity, measuring elasticity of the appendix may provide complementary information in addition to morphologic features on gray-scale US in the diagnosis of appendicitis. The reproducibility of this study needs to be examined in a larger cohort of patients.
